# Supplementary material for: The Increased Amyloidogenicity of Spike RBD and pH-Dependent Binding to ACE2 May Contribute to the Transmissibility and Pathogenic Properties of SARS-CoV-2 Omicron as Suggested by In Silico Study
Source: Int J Mol Sci. 2022 Nov 4;23(21):13502. doi: 10.3390/ijms232113502 (PMC9655063; doi:10.3390/ijms232113502)
Supplement: Supplementary file 1 [file ijms-23-13502-s001.zip › ijms-1882086-supplementary.pdf]

Figure S1. The force reaction in steered Molecular Dynamics simulations of separating the RBD domains from ACE2.

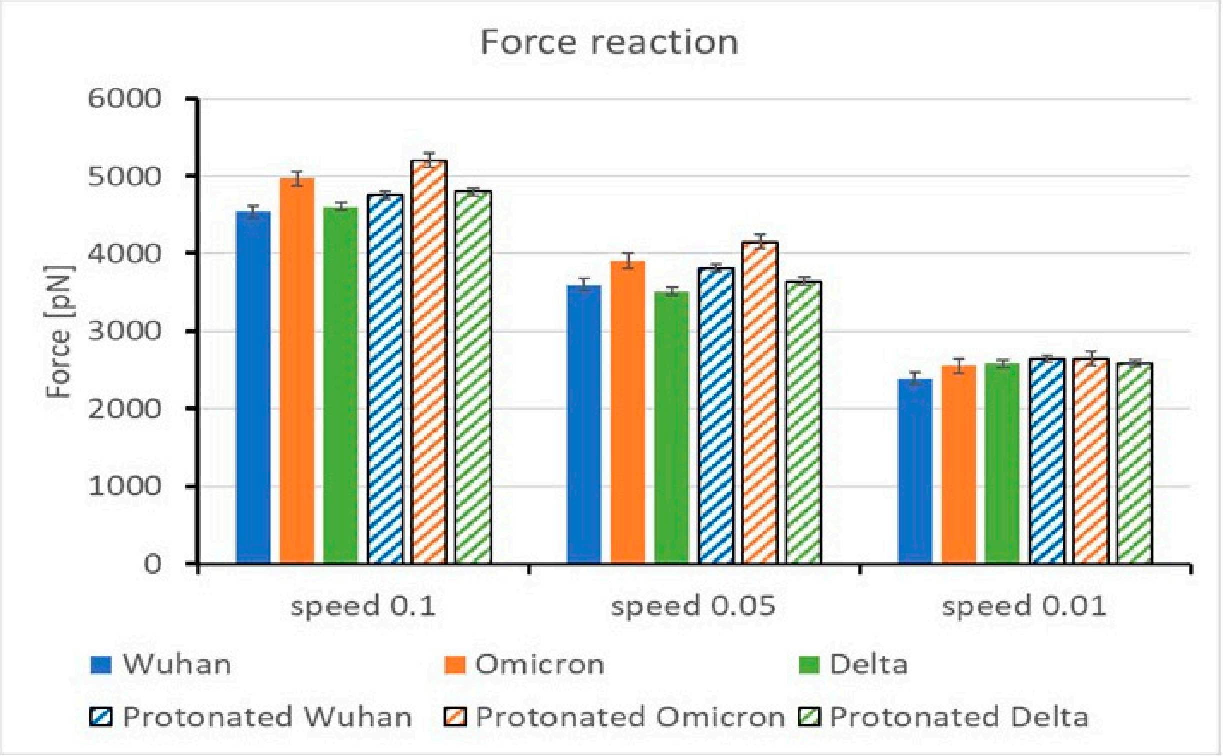

Figure legend: Three constant speeds 0.1, 0.05 and 0.01 Å/ps (indicated below the x-bar) were used to pool away the different Spike RBD variants: Wuhan-Hu-1 (6m0j), Omicron BA.1 (7t9l), Delta (7w9i) and each protonated form (diagonal line pattern) from ACE2. The mean of eight independent experiments is shown in each case, the standard error of the mean is shown as error bars.

Figure S2. Full potential energy of nonvalent bonds of the system after 9ns relaxation

6mj0 (Wuhan-Hu-1 – ACE2)

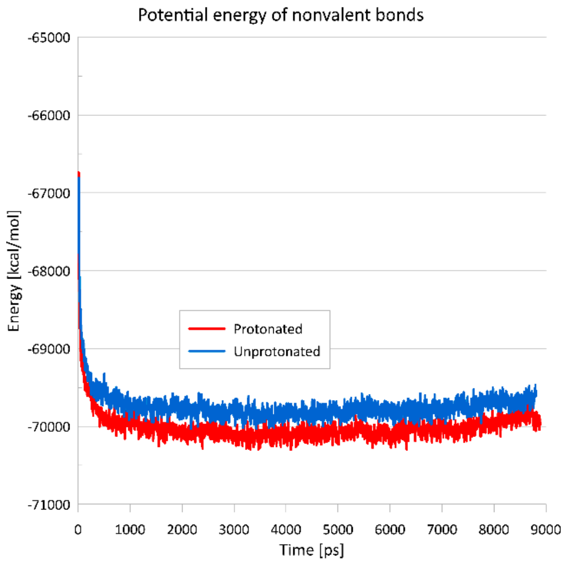

7w9i (Delta – ACE2)

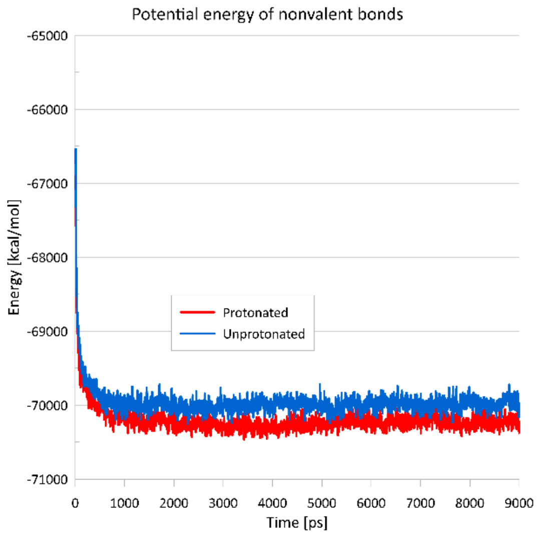

7t9l (Omicron BA.1 – ACE2)

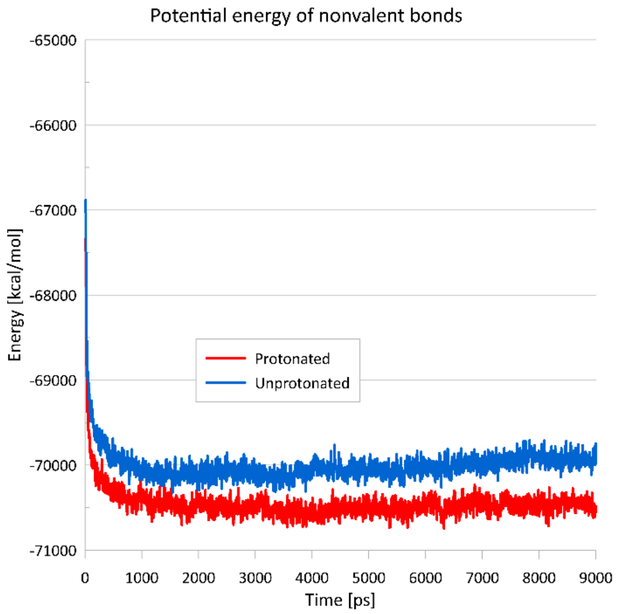

Table S1

Energy of the system in the end of 9ns relaxation.

RBD-ACE2 complex	Unprotonated form [kcal/mol]	Protonated form [kcal/mol]
6m0j (Wuhan-Hu-1)	-69666 ± 142	-69905 ± 147
7w9i (Delta)	-69967 ± 148	-70199 ± 145
7t9l (Omicron BA.1)	-69946 ± 146	-70489 ± 141

Description: The mean of the full potential energy of the system and the standard deviation of the mean for the last 1ns interval is shown.

Figure legend: The full potential energy of non-valent bonding is shown for Wuhan-Hu-1 RBD – ACE2, Delta RBD – ACE2 and Omicron BA.1 RBD – ACE2. Blue corresponds to unprotonated forms, red corresponds to protonated forms. The system includes dissolvent (H_2O), and the full energy is calculated as a sum of Van der Waals and Coulomb interactions.

Figure S3. Force of time dependence for unprotonated forms of RBD and ACE2 in one realization of experiment.

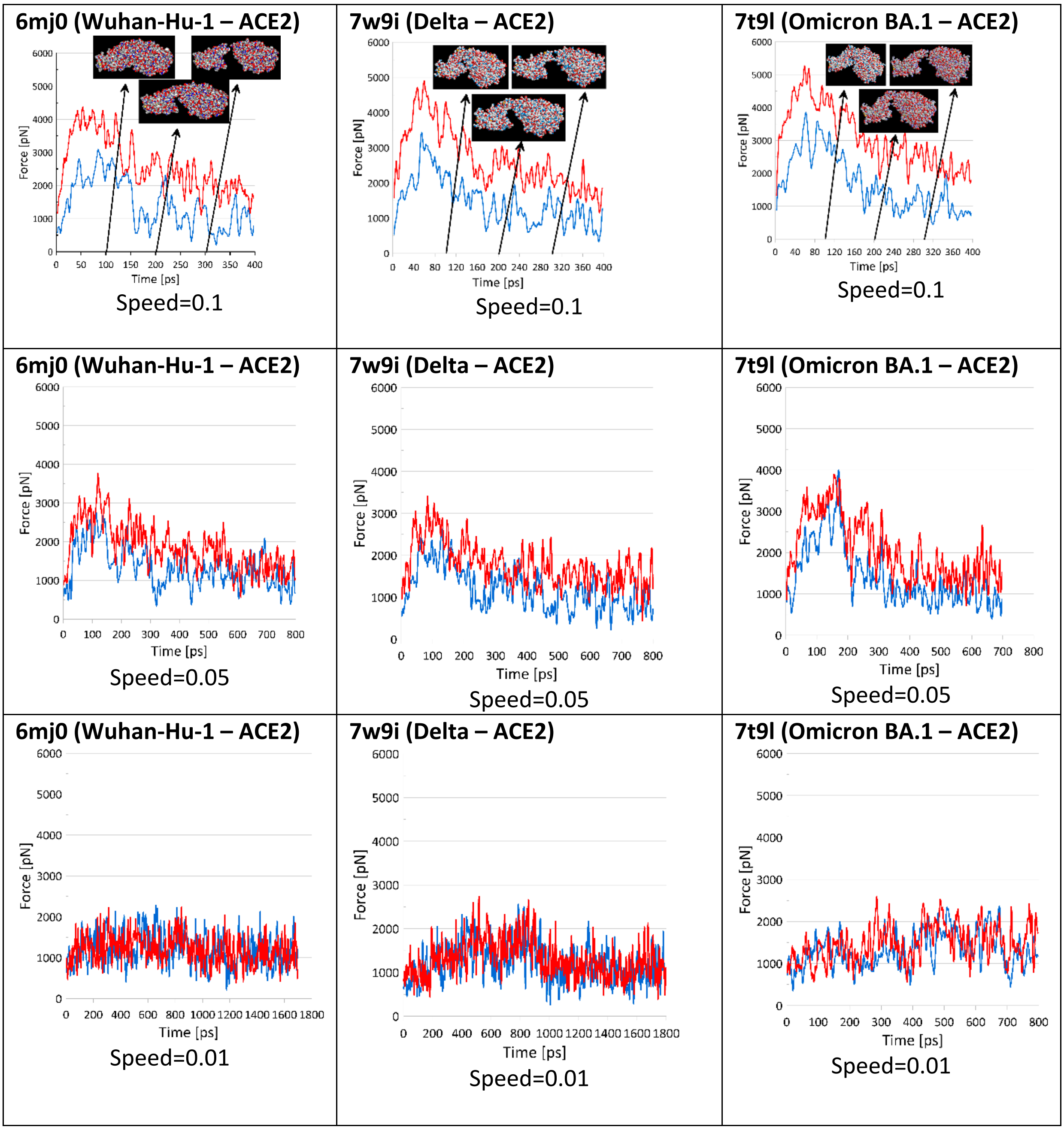

Figure legend: The dependence of force vs time in one realization of pulling RBD from ACE2. The corresponding SARS-CoV-2 RBDs are shown in red, ACE2 is shown in blue. 0.1 Å/ps, 0.05 Å/ps and 0.01 Å/ps speeds are shown.
